# Analysis of amino acids in human tears by hydrophilic interaction liquid chromatography and quadrupole orbitrap mass spectrometry

**DOI:** 10.1039/c9ra05956c

**Published:** 2019-11-11

**Authors:** Chi-Xin Du, Zhu Huang

**Affiliations:** Department of Ophthalmology, The First Affiliated Hospital, College of Medicine, Zhejiang University Hangzhou China flycatty@zju.edu.cn; Department of Ophthalmology, The Fourth Affiliated Hospital, College of Medicine, Zhejiang University Yiwu China

## Abstract

Amino acids in human tears play certain physiological roles and their determination is challenging due to complicated chemical properties. This study described a fast and sensitive method for the simultaneous determination of 15 amino acids (AAs) in tears by hydrophilic interaction liquid chromatography and quadrupole orbitrap mass spectrometry (HILIC-Q-Orbitrap-MS). Amino acids in tears were extracted by methanol, and then cleaned up with a solid phase extraction (SPE) cartridge. Chromatographic separation was performed on a 1.7 μm BEH Amide column within 8 min. Tear samples spiked with free AAs were tested in terms of linearity, sensitivity, repeatability, and recovery. Two stable isotope-labeled amino acids were used as internal standards to improve the method performance. Recoveries for all analytes ranged from 89 to 107%. Intra-day and inter-day precision, expressed as relative standard deviations, were all below 10%, and the method detection limits ranged from 0.02 μmol L^−1^ to 0.11 μmol L^−1^. The developed method with high throughput and high analyte specificity shows good promise for consistent analysis of free amino acids in tears.

## Abstract 2

Amino acids in human tears play certain physiological roles and their determination is challenging due to complicated chemical properties.

## Graphical abstract

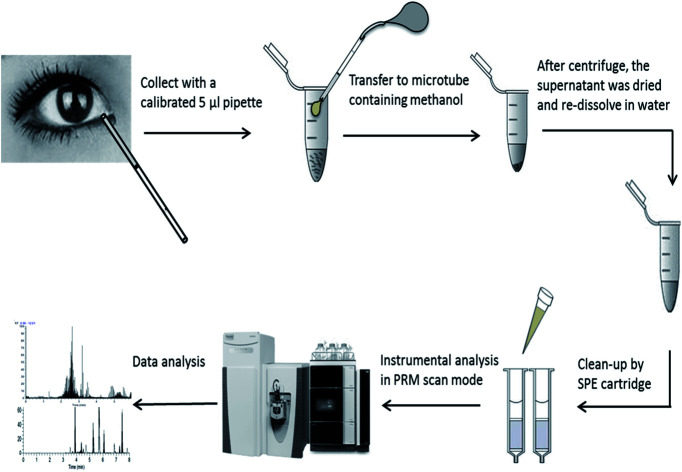

## Introduction

The tear is an extra-cellular fluid bathing the cells of the ocular surface. The profiling of tear metabolites could reflect various physiological processes of surrounding cells. The changes of certain tear metabolites may be associated with ocular surface diseases or potential systemic diseases.^1^ For example, dry eye patients showed a lower concentration of carnitine in tear fluid.^2^ Recently, analysis of amino acids is of particular interest as it also involves the pathophysiology of eye diseases.^3,4^

Amino acids have diverse physical functions. Conventionally, they have long been considered a source of protein synthesis, and play a key role in the regulation of the body metabolism of living organisms. It is now widely accepted that changes in amino acid have profound effects on many aspects of cellular functions, such as gene expression, cell signaling, and the transport of amino acids.^5–7^ Previous reports have pointed out that some amino acids can reduce inflammation by inhibiting NF-κB activation, IL-6 production and expression of the leukocyte adhesion molecule CD62E.^8,9^ Accordingly, exploring amino acids at a trace level in clinical samples prompted the need for a reliable, sensitive and convenient test method.

Previous reports showed that high performance LC (HPLC) with pre- or post-column derivatization and ultraviolet (UV) or fluorescence (FL) detection was the widespread analytical methods for amino acids.^10–12^ But it still remains a challenge to quantify amino acids at trace level in complex biological matrices. More recently, liquid chromatography-tandem mass spectrometry (LC-MS/MS) has been adopted for amino acid analysis in physiological samples due to its excellent selectivity and sensitivity.^13–18^ However, co-eluting endogenous compounds in the matrix interferes with ionization efficiency and reproducibility of the ionization source. To enhance the detection sensitivity and chromatographic retention, derivatization reagents such as *o*-phthaldialdehyde and 9-fluorenylmethyl chloroformate were applied.^15^ However, the methods with chemical derivatization may suffer from laborious sample preparations as well as long testing time.^19,20^ With the development of mass spectrometry (MS) technology, such as orbitrap MS and time of flight (ToF) MS, high resolution MS has become an acceptable method for the direct analysis of amino acids (AAs).^21,22^

Moreover, successful chromatographic separation of amino acids is helpful to the quantification. Usually, reverse-phase high-performance liquid chromatography (HPLC) is used for separation of organic chemicals. However amino acids almost have no retention on normal C18 in analysis of physiological samples due to their high polar and zwitterionic property.^13^ Hydrophilic interaction liquid chromatography (HILIC) is suitable for separation of strongly or moderately polar samples. With highly organic mixed mobile phases, it improves the retention of polar compounds and offers a potential analysis method for amino acids in clinic samples without derivatization or ion-pairing agents. Indeed, usage of HILIC with MS detection to amino acids in biological matrix, as well as clinic samples such as urine, plasma and serum have been successfully applied.^17,18^ However, to our knowledge, few reports refer the analysis of amino acids in human tears through HILIC coupled with high resolution mass spectrometry.^4^

In this study, we developed a liquid chromatography high resolution mass spectrometry method for the simultaneous determination of 15 amino acids (AAs) in human tears. AAs in tears were extracted by methanol and then treated with solid phase extraction (SPE) for clean-up. Separation of AAs was achieved using a HILIC column and detected by Q-Orbitrap mass spectrometry without derivatization. The mode of PRM in Orbitrap instrument was adopted for the quantification of AAs coupled with stable isotope-labeled internal standards.

## Materials and method

### Materials and reagents

A multi-standard solution of amino acids was purchased from Wako Pure Chemistry (Wako Chemical Co., Japan). This multi-standard solution contained l-arginine (Arg), l-aspartic acid (Asp), l-cysteine (Cys), l-glutamic acid (Glu), l-isoleucine (ILe), l-leucine (Leu), dl-lysine (Lys), l-methionine (Met), d-phenylalanine (Phe), dl-proline (Pro), l-serine (Ser), l-threonine (Thr), and dl-valine (Val). The standard solution was stored in darkness at 4 °C. Working standard solutions were prepared daily by diluting the mixed standard stock solution in appropriate proportions. l-Valine-2,3,4,4,4,4′,4′,4-d8, 98 atom% D and l-cysteine-2,3,3-d3, 98 atom% D were purchased from Aldrich, Merck (Darmstadt, Germany). A working solution containing 1 μmol mL^−1^ of each amino acid was prepared in 0.1 M formic acid (FA). All the above solutions were stored at −20 °C until use.

LC-MS grade acetonitrile (ACN) and ammonium formate, ammonium acetate, ammonia, acetic acid and FA from Merck (Darmstadt, Germany) were used to prepare the chromatographic mobile phases. Deionized water from a Millipore Milli-Q water purification system was used to prepare all aqueous solutions. 36.5% (v/v) hydrochloric acid employed for sample preparation was purchased from Merck (Darmstadt, Germany).

### Tear collection

The tear sample was provided by the author of Zhu Huang (female, 38 years old). Basal un-stimulated tears (5 μL) was collected by a calibrated 5 μL pipette (Drummond Science Comp., USA) over 2 min without local anesthesia. The provider had neither ocular complaints nor a history of contact lens usage. The study was approved by Human Research Ethics Committee of the First Affiliated Hospital, College of Medicine, Zhejiang University and performed in accordance with the guidelines dictated by the World Medical Association Declaration of Helsinki of 2013. Informed consents were obtained from human participants of this study. Tear samples (*n* = 6) were obtained triple a day in the morning (a.m. 8:00), noon (a.m. 12:00) and evening (p.m. 8:00). Collected samples were transferred into micro-tube and stored at −80 °C prior to extraction.

### Extraction and clean up

5 μL of each tear sample was extracted by the addition of 95 μL of methanol containing 0.12% formic acid (FA) and mixing with a vortex mixer. The samples were centrifuged for 2 min at 12 000 rpm. The supernatants were then dried by N_2_ flow at 40 °C and re-dissolved in 1 mL water containing 0.2% FA for later clean up.

**Fig. 1:**
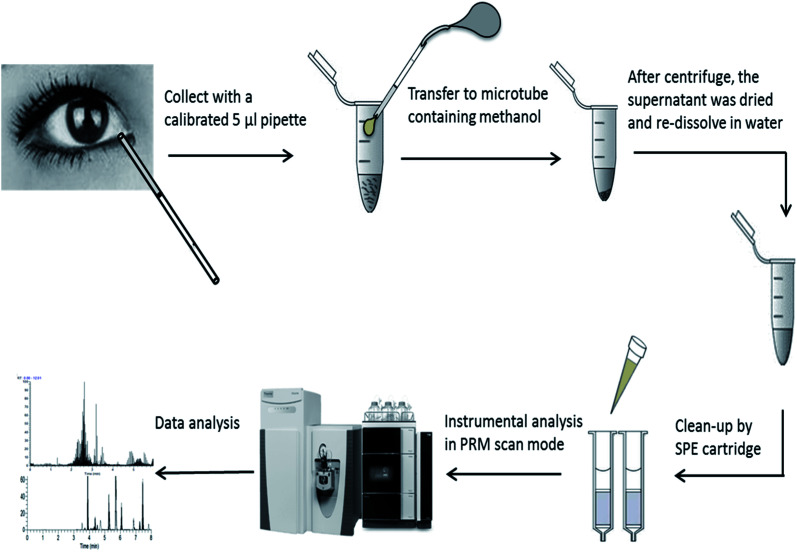
The work flow of analysis of amino acids in human tears by UPLC-Q-Orbitrap-MS.

The extraction was further handled by three different solid phase extraction (SPE) cartridges of HLB, MCX, and WCX (1 cm^3^, 30 mg, Waters Milford, MA, USA). The experimental procedure was based on the suggestions of the supplier and reported methods.^23^ (i) Equilibration: the cartridges were conditioned with 1 mL of methanol followed by 1 mL of water for HLB. For MCX, and WCX, the cartridge was conditioned with 1 mL of methanol followed by 1 mL of acidified water (1% FA). (ii) Loading: acidified samples (0.2% FA) were loaded for HLB, MCX and WCX. (iii) Washing: 1 mL methanol containing 10% water and 0.1% FA was used for all cartridges and repeated at twice. (iv) Elution: 1 mL methanol containing 6% NH_4_OH was used for elution and repeated at twice. Subsequently, eluted solutions were dried by N_2_ flow at 40 °C and re-dissolved in 1 mL water containing 0.2% FA for further instrumental analysis. The performance of SPEs treatment was evaluated by samples spiked with 15 amino acids standard solution at level of 300 μmol L^−1^. Spiking recovery was calculated as: (measure value − matrix value)/spiking value × 100. The whole work flow of AAs analysis can be seen in Fig. 1.

### Instruments and conditions

A Vanquish UHPLC system including a quaternary pump, an autosampler and a column oven was coupled by a HESI-II electrospray source to a Q-Exactive Orbitrap™-based mass spectrometer (all Thermo Scientific, San Jose, CA, USA).

Chromatographic separation was performed on an ACQUITY UPLC BEH Amide column (1.7 μm, 2.1 × 150 mm) (Waters Corporation, MA, USA) at 40 °C. Mobile phase A (50/50 acetonitrile/H_2_O) and B (95/5/5 acetonitrile/methanol/H_2_O) both contained 8 mM ammonium formate and 0.2% formic acid. A gradient elution that started at 90% B for 1 min and decreased linearly to 30%, 1–3 min; 30–60% B, 3–4 min; 60–95% B, 4.0–5.5 min; 95% B, 5.5–6.5 min; 95–90% B, 6.5–8 min. The flow rate was 0.4 mL min^−1^. The sample injection volume was 5 μL.

Q-Orbitrap MS with HESI-II electrospray source was operated in positive mode. The following ionization parameters were applied: electrospray voltage 3.8 kV for positive mode, capillary temperature 340 °C, vaporizer temperature 250 °C, sheath gas (N_2_) 40 arbitrary units (arb), auxiliary gas (N_2_) 15 (arb), and S-Lens RF level at 45 (arb). The instrument was calibrated in positive mode every 7 days using the Pierce LTQ Velos ESI positive-ion calibration solutions (Thermo Scientific, San Jose, CA, USA). The MS parameters of PRM were: default charge 1, inclusion on for monitoring only targeted precursor (Table 1), ms^2^ resolution 17 500, maximum IT 100 ms, AGC target 1.0 × 10^6^, and isolation window 2.0 *m*/*z*. Stepped normalized collision energy (NCE) was 20, 38 and 60. The accurate masses for the precursor are shown in Table 1. The spectra and resulting peaks were manually extracted and evaluated using the Xcalibur software (Version 2.1, Thermo Fischer). Precursor and fragment mass error tolerances were set at 4 ppm and 0.2 Da.

**Table 1:** Mass spectrometry parameters, regression linear range, coefficients of determination (*r*^2^), and limits of detection (LODs)

Comment	Formula [M]	Precursor (*m*/*z*)	Fragment (*m*/*z*)	Retention (min)	Linear range (μmol L^−1^)	*r* ^2^	LODs (μmol L^−1^)
Arginine	C_6_H_14_N_4_O_2_	175.1190	70.0659/116.0709	3.65	0.1–600	0.996	0.05
Aspartic acid	C_4_H_7_NO_4_	134.0448	87.1004/74.0244	3.47	0.1–600	0.995	0.09
Cysteine	C_3_H_7_NO_2_S	122.0270	95.0498/104.0581	2.59	0.1–600	0.996	0.06
Glutamic acid	C_5_H_9_NO_4_	148.0604	84.0451/74.0067	3.14	0.5–600	0.991	0.11
Histidine	C_6_H_9_N_3_O_2_	156.0768	110.0716/103.2544	4.16	0.1–600	0.992	0.08
Isoleucine	C_6_H_13_NO_2_	132.1019	86.0971	1.08	0.1–600	0.995	0.04
Leucine	C_6_H_13_NO_2_	132.1019	86.0971	1.26	0.1–600	0.994	0.02
Lysine	C_6_H_14_N_2_O_2_	147.1128	130.0863	3.99	0.5–600	0.992	0.03
Methionine	C_5_H_11_O_2_NS	150.0583	104.0533/133.0319	1.12	0.1–600	0.993	0.03
Phenylalanine	C_9_H_11_NO_2_	166.0863	120.0810/103.0547	1.05	0.1–600	0.995	0.02
Proline	C_5_H_9_NO_2_	116.0706	70.0659	1.78	0.1–600	0.996	0.04
Serine	C_3_H_7_NO_3_	106.0499	60.0453/88.0400	3.03	0.1–600	0.991	0.06
Threonine	C_4_H_9_NO_3_	120.0655	103.0547/74.0608	2.80	0.1–600	0.992	0.04
Valine	C_5_H_11_NO_2_	118.0863	72.0816	1.59	0.1–600	0.995	0.03
Valine-d8	C_5_H_3_D_8_NO_2_	126.1365	108.1259	1.59	—	—	—
Cysteine-d3	C_3_H_4_D_3_NO_2_S	125.0459	107.0353	2.59	—	—	—

### Method validation

The proposed method was validated in terms of linearity, sensitivity, repeatability, and accuracy. Two stable isotope-labeled AAs were used as internal standard for the compensation of matrix effect. Limits of detections (LODs) for all target compounds were calculated at a signal-to-noise (S/N) ratio of 3. Method accuracy was evaluated by recoveries which were carried out by spiking samples with two different concentrations of standard solutions. Spiking recovery was calculated as: (measure value − matrix value)/spiking value × 100. Intra-day precision was determined by analyzing samples spiked at the same two levels of standards with six replicates, and inter-day precision was determined by running samples with spiked standards at the same levels with three replicates on three different days over a period of 1 week.

### Statistical analysis

All data were analyzed using the analysis of variance (ANOVA) procedure of SPSS statistical analysis software 10.0 (Chicago, IL, USA). As replicated completely randomized designs. A *P* value less than 0.05 was considered statistically significant.

## Results and discussion

### Conditions of Q-Orbitrap-MS

Orbitrap-MS offers high resolution (>20 000 FWHM), accurate mass measurement (<2 ppm), excellent full MS scan sensitivity, and complete mass spectral information. The full MS scan data allow for screening of targeted analytes, confirming positive findings, identifying unknowns or metabolites, and retrospective analysis. Currently, the quadrupole Orbitrap can achieve a high resolving setting for 70 000 or 140 000 FWHM, which ensures highly accurate mass measurements and enables confident discrimination of coeluting, isobaric compounds in complex matrices. Moreover, Q-Orbitrap-MS provides product-ion spectra with accurate mass measurement that permit unequivocal confirmation of compounds of interest.^24,25^ Accordingly, it might be a good choice for the AAs analysis in tears with Q-Orbitrap.

To achieve the highest selectivity and sensitivity, mass spectrometry parameters including ionization mode, capillary voltage, source temperature, sheath gas flow, and collision energy were optimized using AAs standards. The results indicated that most abundant ions (precursor ions) of the AAs were their molecular ions [M + H]^+^ in positive ion mode. The *m*/*z* of precursor ions [M + H]^+^ and characteristic fragment ions for each AA under positive ion mode are listed in Table 1.

PRM scan mode in Q-Orbitrap mass spectrometry is designed for analyte quantification by the MS/MS response. Unlike the MRM of triple quadrupole mass spectrometry, PRM runs with targeted precursor ions screened by quadrupole and transferred *via* the C-trap to the HCD cell for fragmentation (product ions). We found that the stepped value of 20, 38 and 60 of normalized collision energy (NCE) can satisfied the sensitivity of the fragment ions for all AAs. Considering the intensity, the first fragment ion was used for quantification in PRM mode. The intensity was calculated by chromatographic peak area. The balanced dynamic range C-trap setting (1 × 10^6^) was selected to combine the high sensitivity of detection with the extended linear range of quantification. This parameter shows the capacity of any ion trap to control the number of ions. Higher value of the C-trap dynamic range can improve the sensitivity of selected ions through more ion accumulation.

### Optimization of chromatography

**Fig. 2:**
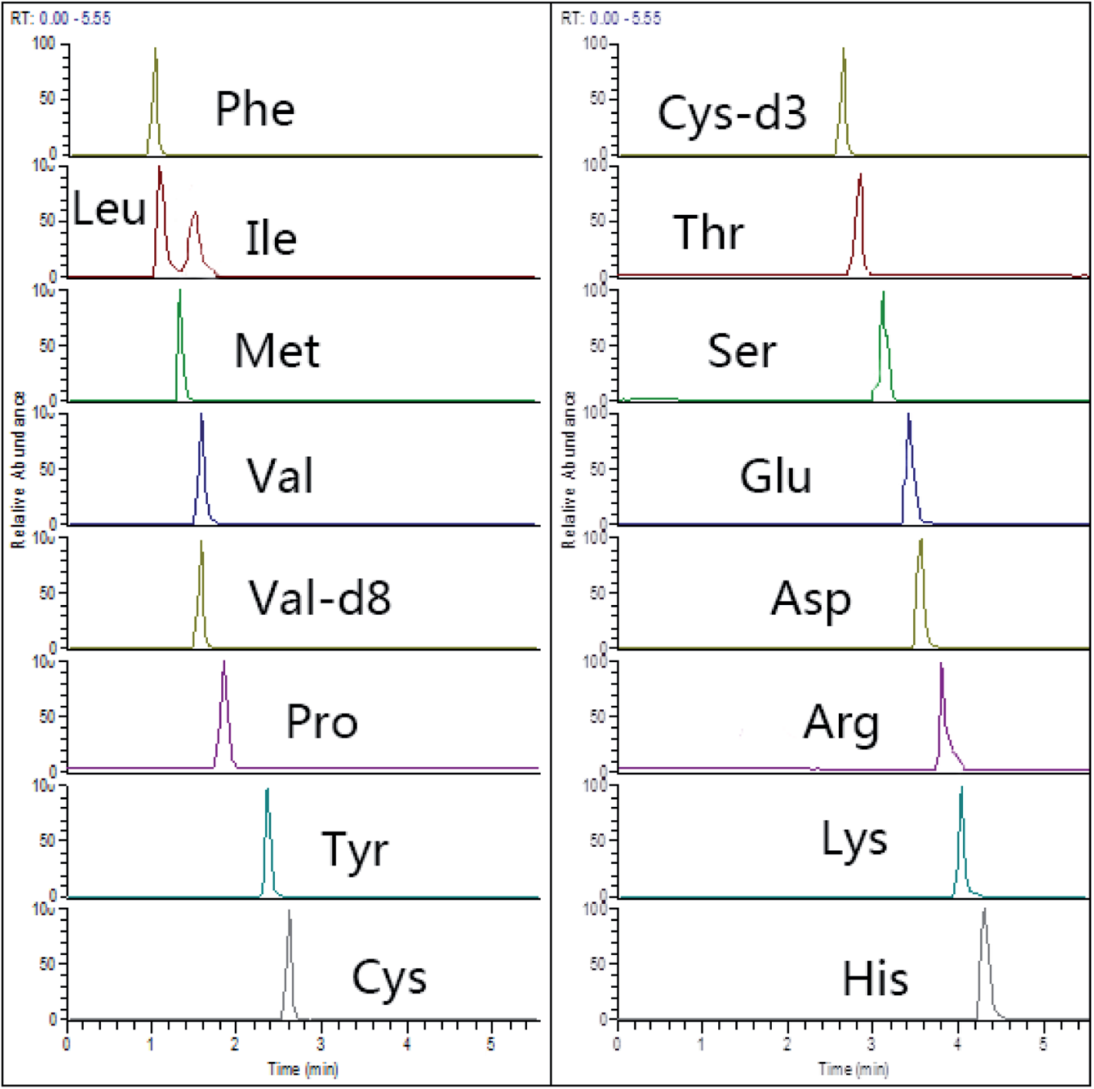
Representative chromatograms of 15 amino acids (spiking level of 20 μmol L^−1^) in HILIC-Q-Orbitrap with PRM mode.

Recently, HILIC columns are gaining popularity due to their simplicity, not requiring traditional derivatization steps or ion-pairing separations for amino acid separation.^26^ We used an ACQUITY UPLC BEH HILIC column (150 mm × 2.1 mm, 1.7 μm) with zwitterionic bonded stationary phase, which showed obviously increased retention of selected AAs (Fig. 2).

To optimize the chromatographic condition, the organic content and buffer concentration in mobile phase were investigated. Similarly to the report of Gao *et al.*,^23^ we found that the increase of buffer concentration made narrow and high peaks of some AAs, such as Asn, Asp, Glu, Lys, Met and His, while it simultaneously decreased MS response (peak height) for other amino acids. The ion suppression or enhancement in mass spectrometry may be produced with the buffer in mobile phase. Moreover, change of buffer concentration between 2 to 10 mM can result in shifting of retention time for some basic or acidic amino acids, such as Arg, Asp, His and Lys. Considering the overall positive effects of higher buffer concentration, especially on Asp and His, we selected a concentration of 8 mM NH_4_HCO_2_ (0.2% FA) as the buffer solution in mobile phase.

### Optimization of sample preparation

The tear fluid is an extremely complicate biological mixture containing proteins/peptides, electrolytes, lipids, and small molecule metabolites. Although most proteins in tears were precipitated by methanol in the extraction step, other components may influence the ionization of the analytes in mass spectrometry. SPE, a well-established technique, has been applied for the analysis of numerous different classes of compounds in a variety of matrices.^27^ As shown in Table 2, three SPE cartridges were compared for their recoveries of spiking samples with 15 amino acids. In terms of the high recovery of 80–98%, we adopted Oasis MCX SPE for further sample treatment. Furthermore, we investigated three elution solvents with varied pH (1.2%, 2%, and 6% NH_4_OH) in MeOH, and it showed that for most of the amino acids, 2% NH_4_OH could elute nearly all analytes. However, with the increasing of eluent pH (6% NH_4_OH), it could increase elution of His, Arg and Lys. Accordingly, 6% NH_4_OH in MeOH was selected as the elution solvent.

**Table 2:** The average recovery of spiking samples with 15 amino acids (spiking level, 300 μmol L^−1^)

Amino acids	Mean recovery (%) (*n* = 5)			RSD (%) (*n* = 5)		
	Oasis HLB	Oasis MCX	Oasis WCX	Oasis HLB	Oasis MCX	Oasis WCX
Arginine	80	89	70	4.5	3.5	5.1
Aspartic acid	82	90	83	3.5	2.8	4.6
Cysteine	90	98	85	2.4	2.5	3.1
Glutamic acid	71	79	70	4.5	3.9	3.4
Histidine	80	85	78	3.7	2.8	3.1
Isoleucine	75	82	73	4.6	5.7	4.1
Leucine	71	80	74	5.9	4.9	4.6
Lysine	85	90	80	7.8	4.2	5.2
Methionine	72	82	71	5.7	3.7	3.9
Phenylalanine	78	82	73	6.5	5.6	5.5
Proline	76	80	71	4.8	4.1	5.3
Serine	70	86	73	6.7	4.6	4.8
Threonine	76	91	75	5.3	3.8	3.9
Valine	88	96	80	2.7	1.9	2.5

### Matrix effect

The matrix effect were evaluated by the spiking the stable isotope-labeled AAs in water and sample matrix. In natural human tears, isotope-labeled AAs (l-valine-d8 and l-cysteine-d3) were not determined. We made a comparison for the mass response of spiked AAs between water and tear samples. The data showed that the intensity of mass response in water was decreased to 70% in tear samples. So, isotope-labeled AAs were adopted as the internal standards spiked in standard solution and samples for the compensation of matrix effects. It should be noted that the use of two isotope-labeled amino acids is less ideal than using all 15 ones.

### Method performance

#### Selectivity

A typical chromatogram of a spiking tear sample is shown in Fig. 2. The separation of AAs was completed by gradient elution in a short time (8 min). No interference was observed at or close to the retention times of AAs and spiking stable internal standards. In addition, no endogenous peaks of the corresponding stable isotope-labeled AAs were observed in human tears. The high selectivity may be ascribed to the accurate mass measurement of high resolution mass spectrometry.

#### Linearity, lower limit of detection, precision, and accuracy

The calibration curve for each analyte was run by using the peak areas of the selected transition obtained at different dilutions (with 0.1 M FA in water) of the stock solution containing all the amino acids. Calibration curves of linear regression model were plotted using analyte/internal standard peak area ratios *versus* concentrations of analytes. The level of spiking internal standards (valine-d8 and cysteine-d3) was 20 μmol L^−1^. Valine-d8 was used as the internal for Phe, Leu, Ile, Met, Val, Pro and Tyr. For Cys, Thr, Ser, Glu Asp, Arg, Lys and His, cysteine-d3 was the internal for calibration. The linear dynamic ranges and regression coefficients are shown in Table 1, with values for the latter above 0.99 for all analytes, and upper limit for calibration dependent on the target compound. The limits of detection (LOD) for each amino acid were calculated as the concentration providing signals three times of background noise. The LODs were ranged from 0.02 μmol L^−1^ for Leu and Phe to 0.11 μmol L^−1^ for Glu. The limits of quantification (LOQ) were defined as the concentration providing signals 10 times of background noise. LOQs were ranged from 0.07 μmol L^−1^ for Leu and Phe to 0.37 μmol L^−1^ for Glu.

Table 3 summarizes the intra- and inter-day precision and accuracy of the method evaluated by spiking samples with different levels. Recoveries of intra- and inter-day tests were 89–107%, and the relative standard deviations (RSDs) were 1.1–5.6%.

**Table 3:** Precision and accuracy from spiking samples of amino acids

Amino acids	Spiking levels (μmol L^−1^)	Intra-day (*n* = 6)		Inter-day (*n* = 3)	
		Recovery (%)	RSD (%)	Recovery (%)	RSD (%)
Arginine	5	93	3.1	91	5.6
	200	95	2.4	96	2.8
Aspartic acid	5	89	2.8	90	4.1
	200	96	2.1	95	3.2
Cysteine	5	95	3.5	94	4.6
	200	99	2.4	102	3.5
Glutamic acid	10	92	3.2	91	4.5
	200	97	2.9	95	3.9
Histidine	5	91	2.5	93	4.2
	200	98	1.8	107	2.4
Isoleucine	5	92	2.4	90	3.4
	200	102	1.9	103	2.8
Leucine	5	95	2.7	94	4.8
	200	105	1.8	104	3.2
Lysine	10	91	3.8	90	4.6
	200	97	2.0	98	2.9
Methionine	5	92	3.6	92	4.5
	200	96	1.7	96	3.1
Phenylalanine	5	96	3.2	91	3.9
	200	106	1.8	99	2.6
Proline	5	91	2.6	93	3.8
	200	97	1.9	102	2.9
Serine	5	92	3.4	95	3.4
	200	95	2.1	98	2.1
Threonine	5	94	3.3	93	4.9
	200	98	1.5	99	2.7
Valine	5	94	3.4	90	3.6
	200	101	1.1	95	2.1

### Application to measurement of amino acids in tears

We further analyzed tear samples (*n* = 6) collected from the provider triple a day in the morning (a.m. 8:00), noon (a.m. 12:00) and evening (p.m. 8:00) using the developed methods. The mean levels of AAs were: arginine (30.2 ± 7.5 μmol L^−1^), aspartic acid (12.5 ± 2.7 μmol L^−1^), cysteine (1.2 ± 0.3 μmol L^−1^), glutamic acid (34.6 ± 6.1 μmol L^−1^), histidine (2.3 ± 0.5 μmol L^−1^), isoleucine (1.1 ± 0.4 μmol L^−1^), leucine (9.2 ± 0.6 μmol L^−1^), lysine (5.8 ± 0.8 μmol L^−1^), methionine (2.9 ± 0.8 μmol L^−1^), phenylalanine (15.4 ± 3.6 μmol L^−1^), proline (2.1 ± 0.9 μmol L^−1^), serine (25.3 ± 4.2 μmol L^−1^), threonine (11.6 ± 2.1 μmol L^−1^), valine (6.9 ± 0.9 μmol L^−1^). Our results were similar with the report of Nakatsukasa *et al.*^4^ where histidine (1.9 ± 0.7 μmol L^−1^), leucine (10.1 ± 1.9 μmol L^−1^), isoleucine (1.1 ± 0.3 μmol L^−1^) were detected. Furthermore, for all tested AAs, no significant difference (*P* > 0.05) among different sample collection periods was found using one-way ANOVA analysis (SPSS 10.0; SPSS, Chicago, IL, USA).

## Conclusion

A fast, sensitive and efficient HILIC-Q-Orbitrap-MS method for the simultaneous determination of 15 amino acids in human tear was developed and validated. By using column of hydrophilic interaction liquid chromatography, strong polar AAs without derivatization could be separated and detected in Q-Orbitrap MS. For reduction of matrix interference, MCX SPE cartridge was successfully applied for sample clean-up. In addition, stable isotope-labeled amino acids used as internal standards could compensate the matrix effect. This method had well precision and accuracy, and could be used for routine analysis of AAs in human tear fluids.

## Author contributions

Z. H. and C.-X. D. conceived the experimental idea at the basis of the study and planned the experiments; Z. H. and C.-X. D. performed analysis of data and wrote the manuscript. All the authors approved the manuscript preparation and submission.

## Conflicts of interest

The authors declare that the research was conducted in the absence of any commercial or financial relationships that could be construed as a potential conflict of interest.

## Supplementary Material
